# Genome-wide association study of blast resistance in indica rice

**DOI:** 10.1186/s12870-014-0311-6

**Published:** 2014-11-18

**Authors:** Caihong Wang, Yaolong Yang, Xiaoping Yuan, Qun Xu, Yue Feng, Hanyong Yu, Yiping Wang, Xinghua Wei

**Affiliations:** State Key Laboratory of Rice Biology, China National Rice Research Institute, Hangzhou, 310006 China; College of Agricultural Sciences, Jiangxi Agricultural University, Nanchang, 330045 China

**Keywords:** Blast disease, Candidate gene, Genome-wide association study, *Oryza sativa* L, *R* protein

## Abstract

**Background:**

Rice blast disease is one of the most serious and recurrent problems in rice-growing regions worldwide. Most resistance genes were identified by linkage mapping using genetic populations. We extensively examined 16 rice blast strains and a further genome-wide association study based on genotyping 0.8 million single nucleotide polymorphism variants across 366 diverse *indica* accessions.

**Results:**

Totally, thirty associated loci were identified. The strongest signal (Chr11_6526998, *P* =1.17 × 10^−17^) was located within the gene *Os11g0225100*, one of the rice *Pia*-blast resistance gene. Another association signal (Chr11_30606558) was detected around the QTL *Pif*. Our study identified the gene *Os11g0704100*, a disease resistance protein containing nucleotide binding site-leucine rich repeat domain, as the main candidate gene of *Pif*. In order to explore the potential mechanism underlying the blast resistance, we further examined a locus in chromosome 12, which was associated with CH149 (*P* =7.53 × 10^−15^). The genes, *Os12g0424700* and *Os12g0427000*, both described as kinase-like domain containing protein, were presumed to be required for the full function of this locus. Furthermore, we found some association on chromosome 3, in which it has not been reported any loci associated with rice blast resistance. In addition, we identified novel functional candidate genes, which might participate in the resistance regulation.

**Conclusions:**

This work provides the basis of further study of the potential function of these candidate genes. A subset of true associations would be weakly associated with outcome in any given GWAS; therefore, large-scale replication is necessary to confirm our results. Future research will focus on validating the effects of these candidate genes and their functional variants using genetic transformation and transferred DNA insertion mutant screens, to verify that these genes engender resistance to blast disease in rice.

**Electronic supplementary material:**

The online version of this article (doi:10.1186/s12870-014-0311-6) contains supplementary material, which is available to authorized users.

## Background

Rice blast disease is a serious and recurrent problem in all rice-growing regions of the world. It is estimated that every year the rice destroyed by the disease could feed 60 million people [1]. The disease is caused by the fungus *Magnaporthe oryzae*, which is the teleomorph of a complex genus of *Ascomycete fungi* composed of interfertile anamorphs [2,3]. The fungus is highly adaptive to its host and is capable of causing infection at any growing stage. The diversity of the pathogen and the complexity of the disease make it to be a formidable challenge for fully solving the problem [1].

The use of resistance (*R*) genes in crop breeding programs has been, and will undoubtedly remain the major means for disease control. In rice, about 180 *R* genes have been isolated to the disease caused by a pathogen infection (http://www.ricedata.cn/ontology). Based on the conserved motifs (nucleotide-binding site (NBS), leucine-rich repeat (LRR), toll-interleukin receptor (TIR), coiled-coil (CC), transmembrance receptor (TM), protein kinase (PK)), *R* genes were classified into four kinds, referring to NBS-LRR, RLK, LRR-TM, TM-CC [4]. More than 68 loci, involved in rice blast resistance, referring to 83 major blast resistance genes, have been identified, and at least 24 resistance genes have been cloned (http://www.ricedata.cn/gene). Blast resistance is generally classified into complete and partial resistance [5]. Complete resistance to blast, controlled by a major gene, is qualitative and race specific, involving genes such as *Pib* [6], *Pita* [7], and *Pi9* [8]. Partial resistance to blast, on other hand, is considered to be quantitative and durable because of its generally non-race-specific and polygenic characteristics. Many partial resistant locus have been identified, such as *pi-21* [9,10]. A rice plant cannot be resistant to an isolate of *Magnaporthe oryzae* unless the pathogen has the genes that make it avirulent on that rice plant. An isolate of *Magnaporthe oryzae* cannot be avirulent on a rice plant unless the rice plant has genes that make it resistant to that isolate [11]. Hence, cultivating rice varieties with highly efficient, durable resistance to blast is still the most economically feasible and environmentally sound management approach in most blast-prone rice ecosystems.

The QTL approach, specific to the genetic population, is not suitable to identify the tremendous phenotypic variation within the scope of the whole genome [12,13]. The genome-wide association study (GWAS) has emerged as a powerful approach for simultaneously screening genetic variation underlying complex phenotype. In 2005, GWAS was first applied to a human disease, age-related macular degeneration [14]. Subsequently, a series of GWAS research have been published [15-18]. However, GWAS applied to the dissection of complex traits in animals and plants are only just beginning because of the lack of effective genotyping techniques and the limited resources for developing high-density haplotype maps. For both QTL approach and GWAS, genetic transformation is generally required to identify the candidate gene(s).

In rice, increasing amounts of genomic resources have been created in terms of genome sequences [19,20] and high-density SNP maps [12,21,22]. Huang et al.[21,22] used Illumina next-generation sequencing of abundant rice landraces worldwide to generate low-coverage sequence data across the lines and construct a comprehensive HapMap for rice (*Oryza sativa*) that could be used for GWAS for agronomic traits. In our study, we extensively examined blast resistance in a genome-wide association study (GWAS) based on genotyping 805,158 SNPs variants across 366 *indica* diverse accessions [21,22]. The goal of this study was, using GWAS, to identify a substantial number of loci related to blast resistance that could be important for rice production and improvement.

## Results

### Phenotypic variation

We investigated the concentrations of 16 strains of rice blast on seedlings, and evaluated resistance to rice blast by DLA on each experimental plot. The correlation coefficient indicated that DLA by blast strains CH131, CH154 and CH159 was significantly associated with latitude (respectively *r* =0.12, *P* =2.4 × 10^−2^; *r* =0.15, *P* =3.4 × 10^−3^; *r* =0.14, *P* =6.9 × 10^−3^) while DLA by CH251 was associated with longitude (*r* = −0.27, *P* =1.6 × 10^−7^) (Additional file 1: Figure S1). Based on the SharedAllele distance [23] using the unweighted pair-group method with arithmetic mean (UPGMA) [24], the set of strains could be divided into two groups: one group only consisted of the strains CH131, CH212, CH362 and CH193, which have the weaker pathogenicity, and the other comprised the remaining 12 strains, which have the stronger pathogenicity (Additional file 2: Figure S2).

### GWAS for resistance to 16 blast strains

**Figure 1:**
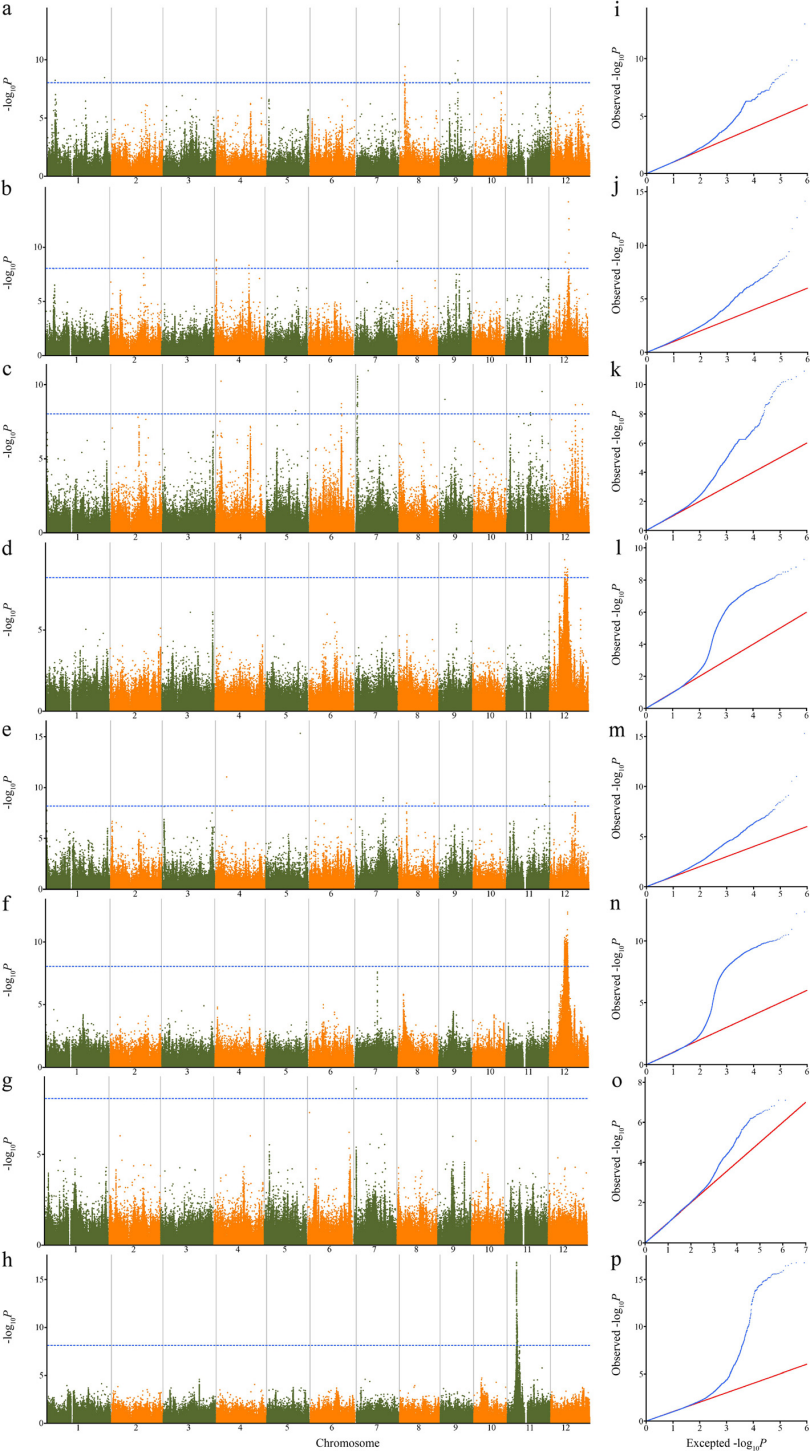
**Genome-wide association studies of rice blast resistance.** Manhattan plots for eight strains, **(a)** CH102, **(b)** CH149, **(c)** CH171, **(d)** CH172, **(e)** CH182, **(f)** CH186, **(g)** CH212, **(h)** CH362. Negative log10-transformed *P* values from a genome-wide scan are plotted against position on each of 12 chromosomes. Gray horizontal dashed line indicates the genome-wide significance threshold. Quantitle-quantitle plot for the eight strains, **(i)** CH102, **(j)** CH149, **(k)** CH171, **(l)** CH172, **(m)** CH182, **(n)** CH186, **(o)** CH212, **(p)** CH362.

To investigate the genotypic variation underlying resistance to rice blast, GWAS was carried out to identify the associated loci in *indica* rice landraces, using the EMMAX algorithm [25]. We identified a total of 30 associated loci using *P* =1.0 × 10^−8^ as the genome-wide significance thresholds (Figure 1, Table 1). 50% of the detected strains (8 out of 16) had at least one significant association, with an average of 3.8 associations per strains. The CH171 strain had most with eight associations, followed by CH182 with seven, while CH186, CH212 and CH362 only had one. Genome-wide analysis of the associated loci existed random distribution across the 12 chromosomes. Of these loci, most seven were separately distributed on chromosome 11 and chromosome 12, while none were on chromosome 10. The chromosome distributions of the associated loci are presented in Additional file 3: Figure S3, possessing similar trends with the distributions of the known loci. Furthermore, GWAS hot spots were located on chromosome 11 and 12, which was consistent with the reported research [26]. And the same/nearby SNPs were significantly associated with multiple traits. For example, SNPs at 30.6 Mb on chromosome 11 were associated with CH182 and CH149, SNPs at 13.0 Mb on chromosome 12 with CH172 and CH186. This illustrated that these strains might have common mechanisms and be caused by pleiotropic or closely linked genes. The significant loci detected are also illustrated, corresponding to disease resistance protein, NBS-LRR protein, kinase-like domain containing protein, heavy metal transport/detoxification protein and other known and unknown proteins (Additional file 4: Figure S4).

**Table 1:** **Genome-wide significant association signals of rice blast resistance using the EMMAX algorithm**

**Strain**	**Chromosome**	**Position**	**Allele**	**MAF** ^**a**^	***P*** **-value**	**Known loci**
CH102	1	5686415	T, A	0.068	6.14E-09	
CH102	8	3774060	C, T	0.063	4.36E-10	
CH102	9	10663921	G, A	0.053	1.65E-09	
CH102	11	21585376	G, A	0.057	2.97E-09	
CH149	2	24129162	C, T	0.060	1.04E-09	
CH149	4	1193240	C, T	0.120	1.51E-09	
CH149	4	24398518	A, C	0.065	5.06E-09	
CH149	9	12562701	C, T	0.078	1.30E-10	
CH149	11	30618466	G, A	0.102	9.94E-09	*Pif* [29]
CH149	12	13690289	C, A	0.060	7.53E-15	
CH171	4	3571241	G, A	0.068	6.28E-11	
CH171	5	22378428	A, T	0.063	3.27E-10	
CH171	6	22777049	C, T	0.057	2.05E-09	
CH171	7	1181446	A, G	0.052	2.95E-11	
CH171	11	16829017	A, G	0.122	8.00E-09	
CH171	11	25107794	T, A	0.065	3.09E-10	
CH171	12	18051954	C, T	0.058	2.46E-09	
CH171	12	23040661	G, A	0.089	2.33E-09	
CH172	12	10833734	C, A	0.469	5.33E-10	
CH172	12	12959480	G, A	0.467	1.98E-09	
CH182	3	1170958	A, G	0.060	7.92E-09	
CH182	7	19794146	T, A	0.070	1.16E-09	
CH182	8	5587272	C, T	0.055	3.77E-09	
CH182	8	25225491	C, T	0.055	3.87E-09	
CH182	11	27068156	C, T	0.300	5.36E-09	*Pik* [28]
CH182	11	30606558	C, T	0.060	2.94E-11	*Pif* [29]
CH182	12	17934412	A, G	0.076	2.85E-09	
CH186	12	13032951	G, A	0.433	4.25E-13	
CH212	7	1616159	C, T	0.172	2.40E-09	
CH362	11	6526998	G, A	0.240	1.72E-17	*Pia* [27]

^a^MAF: minor allele frequency.

### Assessment of GWAS findings and function identification of candidate genes

Searching the flanking regions of the associated loci, four were located close to or even landed on two known cloned genes (*Pia* [27], *Pik* [28]), and one QTL (*Pif* [29]), identified previously using near isogenic lines or recombinant inbred lines with map-based cloning, which illustrated the relatively high resolution of our GWAS. We forecasted candidate genes through searching a protein that contained the conserved motifs of *R* gene. To further verify the association possibility, we validated some of candidate genes by quantitative real-time PCR (*q*PCR) (Additional file 5: Table S1, Additional file 6: Table S2) and their expression profiles in public databases.

**Figure 2:**
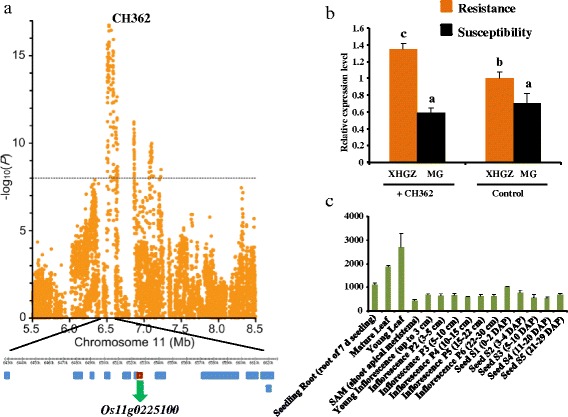
**Associations and genomic locations of known gene,** ***Os11g0225100*** **. (a)** The strongest signal located in the coding region. **(b)** Comparisons of expression before and after inoculation. **(c)** The expression pattern of *Os11g0225100* from public microarray data.

*R* genes play vital part in the detection of invading pathogens, and in the activation of defense mechanisms [30], among which NBS-LRR-type *R* genes have been the most extensively studied research targets in plant genetics. The strongest association result (peak SNP: Chr11_6526998; *P* =1.17 × 10^−17^), explaining up to 26.6% of the phenotypic variance, was around the gene *Os11g0225100* (Figure 2a), which is one of the rice *Pia*-blast resistance gene and encodes NBS-LRR type protein from a region on chromosome 11 [27]. The expression of *Os11g0225100* (Figure 2b) in the resistant landrace was higher than that in the susceptible landrace. After inoculation, the resistance level in the resistant landrace increased, while it has no change in the susceptible landrace. The expression profiles from microarray data (Figure 2c) indicated that the *Os11g0225100* gene has a high expression level in young leaf, mature leaf and seeding root.

**Figure 3:**
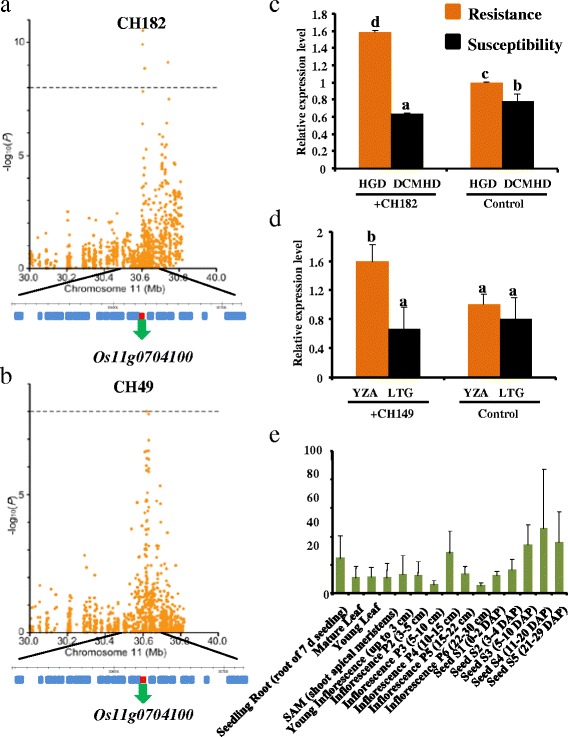
**The strong associated signal near previously identified QTL,** ***Pif*** **. (a)** Association with CH182. **(b)** Association with CH149. Comparisons of expression before and after inoculation. **(c)** Inoculation with the strain, CH182. **(d)** Inoculation with the strain, CH149. **(e)** The expression pattern of *Os11g0704100* from public microarray data.

We observed additional signals located ±13 kb and 0.5 kb downstream of the *Pif*, a rice blast disease QTL identified by a previous study [29] and mapped on chromosome 11. These SNPs were associated with CH182 (Chr11_30606558, *P* =2.94 × 10^−11^, Figure 3a) and CH149 (Chr11_30618466, *P* =9.94 × 10^−9^, Figure 3b), respectively explaining 13.6% and 15.9% of the phenotypic variance. *Os11g0704100* is described as a disease resistance protein containing nucleotide-binding and leucine-rich repeat (NB-LRR) domain, suggesting that *Os11g0704100* is the largest extent candidate gene for *Pif*. As shown in *q*PCR (Figure 3c,d), the expression of *Os11g0704100* was similar to that of *Os11g0225100*. The expression profiles from microarray data (Figure 3e) showed that the gene was constitutively expressed at a low level. Thus, we speculated that the disease resistance protein *Os11g0704100* might be the large extent candidate gene for *Pif*.

**Figure 4:**
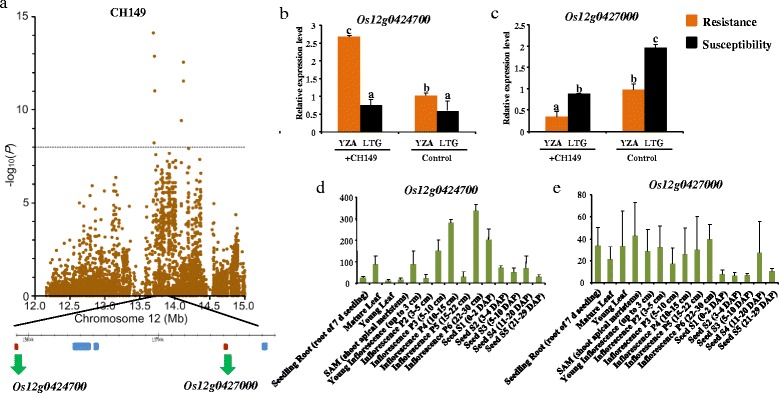
**New regions resulting from GWAS. (a)** Top of panel shows a 150 kb region on each side of the peak SNP, Chr12_13690289. Comparisons of expression before and after inoculation. **(b)** Inoculation with the strain, CH182. **(c)** Inoculation with the strain, CH149. **(d)** The expression pattern of *Os12g0424700* from public microarray data. **(e)** The expression pattern of *Os12g0427000* from public microarray data.

Members of the kinase protein family also participate in *R* gene-mediated disease resistance, such as the reported genes *Pto* (in tomato) [31], *Xa21* (in rice) [32], and *Rpg1* (in barley) [33]. A significant SNP (Chr12_13690289, *P* =7.53 × 10^−15^, Figure 4a) associated with CH149 for a cluster of six genes (Table 2), explaining 18.0% of the phenotypic variance. According to gene ontology (GO) analysis, *Os12g0424700* and *Os12g0427000* were described as kinase-like domain containing protein and identified as the *priori* candidate genes underlying this locus. For *Os12g0424700*, the result of *q*PCR (Figure 4b) was also similar to *Os11g0225100*. And the expression profiles from microarray data (Figure 4d) indicated that *Os12g0424700* has a low expression level, with a peak during inflorescence P6 (22-30 cm). For *Os12g0427000*, the expression level decreased after inoculation (Figure 4c) and it has a low expression level in most tissues and organs (Figure 4e), similar to *Os11g0704100*. Therefore, we speculated that the two genes might be required for the full function of this locus. This is similar to the situations in rice *Pikm* [34], *Pi5* [35], and *Arabidopsis RRS1* and *RPS4* [36], which demonstrated that the exact same phenotype of complete disease resistance can be the result of different loci.

**Table 2:** **Summary of six candidate genes for Chr12_13690289**

**Candidate gene**	**Annotation description**
*Os12g0424700*	Protein kinase-like domain containing protein.
*Os12g0425500*	Non-protein coding transcript/uncharacterized transcript
*Os12g0425600*	Growth regulator related protein
*Os12g0425800*	Hypothetical protein
*Os12g0427000*	Protein kinase-like domain containing protein
*Os12g0427600*	Proteinase inhibitor I9 subtilisin propeptide domain containing protein

**Figure 5:**
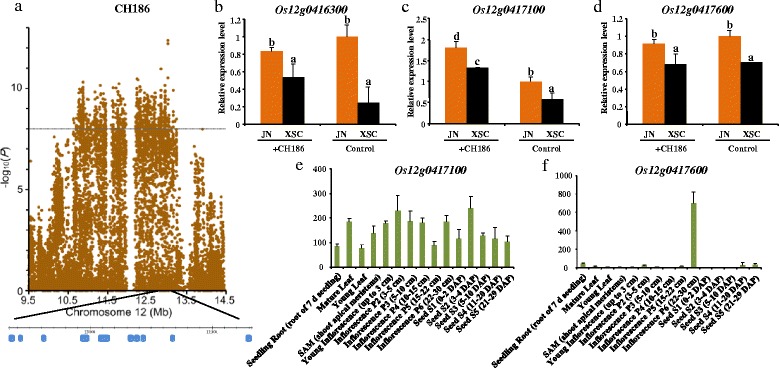
**Novel functional loci test by GWAS. (a)** The associated locus, Chr12_13032951. The expression before and after inoculation of genes, **(b)** *Os12g0416300*, **(c)** *Os12g0417100* and **(d)** *Os12g0417600*. **(e)** The expression pattern of *Os12g0417100* from public microarray data. **(f)** The expression pattern of *Os12g0417600* from public microarray data.

To evaluate whether novel functional loci were implicated by GWAS, we further explore significant SNP, Chr12_13032951 (*P* =4.25 × 10^−13^), on chromosome 12. This SNP was significantly associated with the strain CH186 (Figure 5a), and explained up to 21.6% of the phenotypic variance. Searching the flanking region, ten candidate genes were involved, referring to zinc finger protein, histone H3, putative plant transposon protein and so on (Table 3). Our qPCR analysis (Figure 5b,c, Additional file 7: Figure S5) demonstrated that *Os12g0416300*, *Os12g0417100*, and *Os12g0417600* might be the most promising candidate genes participated positive regulations for this region. *Os12g0416300* (Figure 5b) and *Os12g0417600* (Figure 5d) had a higher expression levels in the resistant landraces compared with those in the susceptible landraces, and no difference between before and after inoculation. Unlike them, the expression level of *Os12g0417100* (Figure 5c) increased after inoculation. In addition, the microarray data showed that *Os12g0417100* (Figure 5e) were constitutively expressed at a low level, *Os12g0417600* (Figure 5f) had a high expression level during the inflorescence P6 (22–30 cm) and weak expression levels in other tissues and organs at various development stages. There were no probes in the microarray for *Os12g0416300*. For others, they might be no role in the regulation of blast resistance or as negative regulation (Additional file 8: Figure S6).

**Table 3:** **Summary of ten candidate genes for Chr12_13032951**

**Strain**	**Associated loci**	**Candidate gene**	**Description**
CH186	Chr12_13032951	*Os12g0414900*	Zinc finger RanBP2-type domain containing protein
CH186	Chr12_13032951	*Os12g0415400*	Similar to Histone H3
CH186	Chr12_13032951	*Os12g0415800*	Similar to Histone H3
CH186	Chr12_13032951	*Os12g0416300*	Conserved hypothetical protein
CH186	Chr12_13032951	*Os12g0416500*	GCN5-like 1 family protein
CH186	Chr12_13032951	*Os12g0416800*	Hypothetical protein
CH186	Chr12_13032951	*Os12g0416900*	Conserved hypothetical protein
CH186	Chr12_13032951	*Os12g0417000*	Putative plant transposon protein family protein
CH186	Chr12_13032951	*Os12g0417100*	Non-protein coding transcript unclassifiable transcript
CH186	Chr12_13032951	*Os12g0417600*	Non-protein coding transcript putative npRNA

Of the other SNPs reaching genome-wide significance, Chr11_27068156 (*P* =5.36 × 10^−9^) was ±37 kb upstream of the known cloned gene, *Pik*, which is also composed of two adjacent NBS-LRR genes and confers high and stable resistance to many Chinese rice blast isolates [28]. More interesting, we discovered a signal (Chr03_1170958, *P* =7.92 × 10^−9^, Figure 1e, m) associated on Chromosome 3, where there is no relative blast resistance loci reported. Searching the flanking region (Additional file 9: Table S3), *Os03g0122000* encodes kinase-like domain containing protein, and *Os03g0120400* encodes heavy metal transport/detoxification protein domain containing protein, and so on. Though it is unclear what gene participate the resistance regulation, the region would be further investigated.

## Discussion

In this work, GWAS was used for association mapping of quantitative disease resistance genes to rice blast disease, which is similar to work performed in maize [37]. The use of high-density genome-wide SNPs in GWAS not only allows the discovery of true candidate genes, but also enables a comprehensive view of the regulatory mechanism of the traits.

Although genome-wide association studies are becoming more and more feasible, it seems likely that population structure will still be subject to considerable debate, which may result in an increase rate of the false-positives [38]. However, the extent of the problem not only depends on the extent to that the sample is structured, but also on the phenotype [39]. A trait that is strongly affected by population structure will display a higher false-positive rate [40-42]. In order to be less prone to false-positives resulted from genetic structure, our study only used the *indica* panel and optimized all of the parameters in the EMMAX algorithm. In fact, although the EMMAX algorithm, or mixed line model [43], reduced the inflation of the *P*-value, it could often mask the true loci and decrease the detection power [12]. In this context, most association mapping of human studies are likely to be case–control studies, given a judiciously chosen control [44].

Despite these limitations, we identified 30 loci associated with rice blast disease resistance in the *indica* panel, with one located on chromosome 3. It is no doubt that there is pseudo information in the data, and not practical to thoroughly predict the false association from true. To analyze the candidate genes, we combined gene annotation, *q*PCR and expression profile from microarray data to investigate the potential functional polymorphisms capable of causing changes in the phenotype. Of these associated loci, some were overlapped or coincided with previously identified genes and QTLs [27-29]. If the range was extended to 300 kb (±150 kb), more known genes might have been detected, such as *Pita* [7,45,46]. In addition, we detected newly associated loci that were characterized by the presence of *R* genes. Furthermore, it is worth noting that the strongest association did not always correspond to the candidate genes, which might reflect an ascertainment bias [12], and these genes may be more interesting because of their participation in metabolic regulation [38]. The results demonstrated that rice blast disease resistance was conditioned by a range of mechanisms [47], and that there is considerable mechanistic overlap with basal resistance [48].

Chromosomal hotspots are frequently found for rice blast disease, and chromosome 11, as previously reported, had the most associated loci: 15 loci referring to 24 major blast resistance genes (http://www.ricedata.cn/gene). In this study, associated loci and candidate genes were also frequently found on chromosome 11. Yu *et al*. [49] discovered that the highest frequency of copy number variations (CNVs) for rice was on chromosome 11, and genes in many CNVs were involved in resistance. Most of these encode proteins with conserved nucleotide-binding sites (NBS) and leucine-rich repeats (LRRs). Meanwhile, NBS-LRR genes in plants are inclined to cluster at the adjacent loci within genomes [50]. We also analyzed a cluster of candidate genes that cooperatively participated in functional regulation of blast disease resistance, or had a direct role in regulation , as observed in the a previous study [28]. The explanation to the hotspots might be the occurrence of biochemical connections or that they are highly related with the original rice genome, pointing to the same genomic position.

We found that several SNPs were associated with the same strain. And this might be multigenic effect and attributed to the accumulation of numerous loci, influenced by epistatic effect and additive effect. In addition, some strains showed a significant association in the same region, indicating that these strains had similar genetic control, moreover, illustrating that these strains might have common mechanism and be caused by pleiotropic or closely linked genes [51]. This result was in line with classification according to the phenotype data. These correlations indicated that the mutation in this identified region was an important control point for blast disease and should be considered as a quantitative partial resistant to blast because of its generally non-race specificity. Otherwise, the different *P* values of these correlations demonstrated that the loci may reflect unequal disease resistance for the various strains.

## Conclusions

The use of high-density genome-wide SNPs in GWAS not only allows the discovery of true candidate genes, but also enables a comprehensive understanding of the regulatory mechanism of the traits. Our results further confirmed that GWAS is a powerful complementary approach for dissecting the quantitative disease resistant genes to traditional QTL mapping. This work provides the basis of further study of the potential function of these candidate genes. A subset of true associations would be weakly associated with outcome in any given GWAS; therefore, large-scale replication is necessary to confirm our results. Future research will focus on validating the effects of these candidate genes and their functional variants using genetic transformation and transferred DNA insertion mutant screens, to verify that these genes engender resistance to blast disease in rice.

## Methods

### Plant materials

The association mapping panel we used was composed of 517 Chinese rice landraces previously described in detail by Huang et al. [22] and deposited in the EBI European Nucleotide Archive(Accession numbers ERP000106), which included the *indica* panel (366 *indica* varieties) and the *japonica* panel (136 *japonica* varieties). Given the strong population differentiation between the two subspecies of cultivated rice, we did not look for associations across the entire set. Meanwhile, due to the less sample size of the *japonica* panel, we carried out GWAS for the subset of *indica* rice.

### Phenotypic variation

Phenotypic measurements were obtained at the China National Rice Research Institute Farm in Hangzhou, China at N 30°32', E 120°12' in 2012. Seeds were planted in plastic trays (43*30*7.5 cm) to test blast resistance for 16 strains (CH102, CH122, CH131, CH149, CH154, CH159, CH171, CH172, CH182, CH186, CH193, CH212, CH218, CH242, CH251, CH362) represent collected from all over China. Fifteen seeds were planted in each of six rows and ten ranks per tray with three replications. Plants were incubated at the third-to-fourth leaf stage by the spraying method in low light and at room temperature as well as high humidity (between 70 and 85%) to ensure sporulation and subsequent reinfection of susceptible plants. The disease reactions were measured about 7 d after inoculation, and evaluated by diseased leaf area (DLA) [52]. Higher DLA among replications was used in the analysis.

### Genotyping and association mapping

We used the sequencing data of Huang et al. [21,22] in the Rice Haplotype Map Project Database (http://www.ncgr.ac.cn/RiceHap2). SNPs with a minor allele frequency >5% were used for the association analyses. We used the Efficient Mixed-Model Association eXpedited (EMMAX) algorithm to carry out the GWAS [25].

### Analysis of significant signals

To identify candidate genes and predict their putative functions in the associated loci for the corresponding strain, we used gene annotation information from the Rice Annotation Project Database (RAP-DB). All potential candidate genes in the associated loci, which had specific roles in rice blast resistance responses, were selected within a 200-kb genome region (100 kb upstream and 100 kb downstream of the peak SNPs).

### Identification of the candidate genes

The expression levels of the candidate genes before and after blast disease infection were measured using quantitative real-time PCR (*q*PCR). Total RNA was extracted from young rice leaves using an AxyPrep™ Multisource Total RNA Miniprep Kit from Axygen (Tewksbury, MA, USA). Complementary DNA (cDNA) was synthesized with a dT18 primer from total RNA using the First Strand cDNA Synthesis Kit from Toyobo Co. (Osaka, Japan). Quantitative real-time PCR (*q*PCR) primers (Additional file 5: Table S1) and materials (Additional file 6: Table S2) for amplification were designed, and the reaction was performed on a 7500 Real-Time PCR system (Applied Biosystems, Carlsbad, CA, USA). The expression level of *β*-actin was used to standardize the RNA sample for each analysis. The *q*PCR assay was performed at least three times for each experimental line. The expression profile analyses were also performed using the database in the Bio-Array Resource for Plant Biology (http://bar.utoronto.ca).

## Additional files

Additional file 1: Figure S1 Geographical distribution of phenotypic variation for 16 strains. Red dots indicate resistance; green dots indicate moderate susceptibility; black dots indicate susceptibility. Additional file 2: Figure S2 UPGMA dendrogram of 16 strains based on the SharedAllele distance. Additional file 3: Figure S3 The number of the associated loci by GWAS in each chromosome. Additional file 4: Figure S4 Functional category annotation for the associated loci by GWAS. Additional file 5: Table S1 Primers used for quantitative real-time PCR. Additional file 6: Table S2 Materials used for quantitative real-time PCR. Additional file 7: Figure S5 Quantitative real-time PCR analysis of the candidate genes for the associated locus, Chr12_13032951. Additional file 8: Figure S6 Expression pattern analysis of the candidate genes for the associated locus, Chr12_13032951. Additional file 9: Table S3 Summary of 20 candidate genes for Chr03_1170958.
