# Supplementary figures and images for: Genome-wide association study of blast resistance in indica rice
Source: BMC Plant Biol. 2014 Nov 18;14:311. doi: 10.1186/s12870-014-0311-6 (PMC4239320; doi:10.1186/s12870-014-0311-6)

## Slide 1
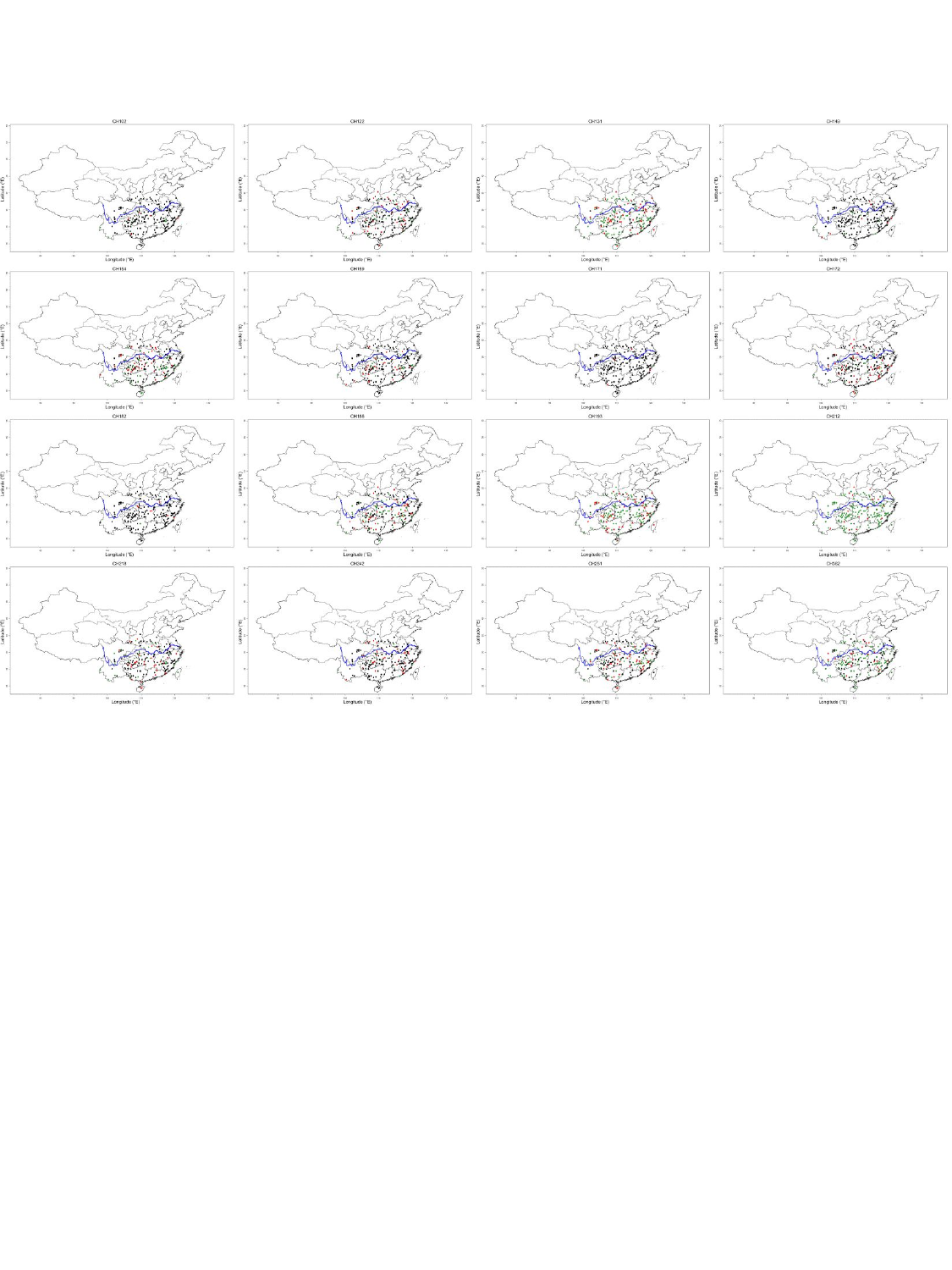

Supplement: Additional file 1: Figure S1 — Geographical distribution of phenotypic variation for 16 strains. Red dots indicate resistance; green dots indicate moderate susceptibility; black dots indicate susceptibility. [file 12870_2014_311_MOESM1_ESM.ppt]

## Slide 1
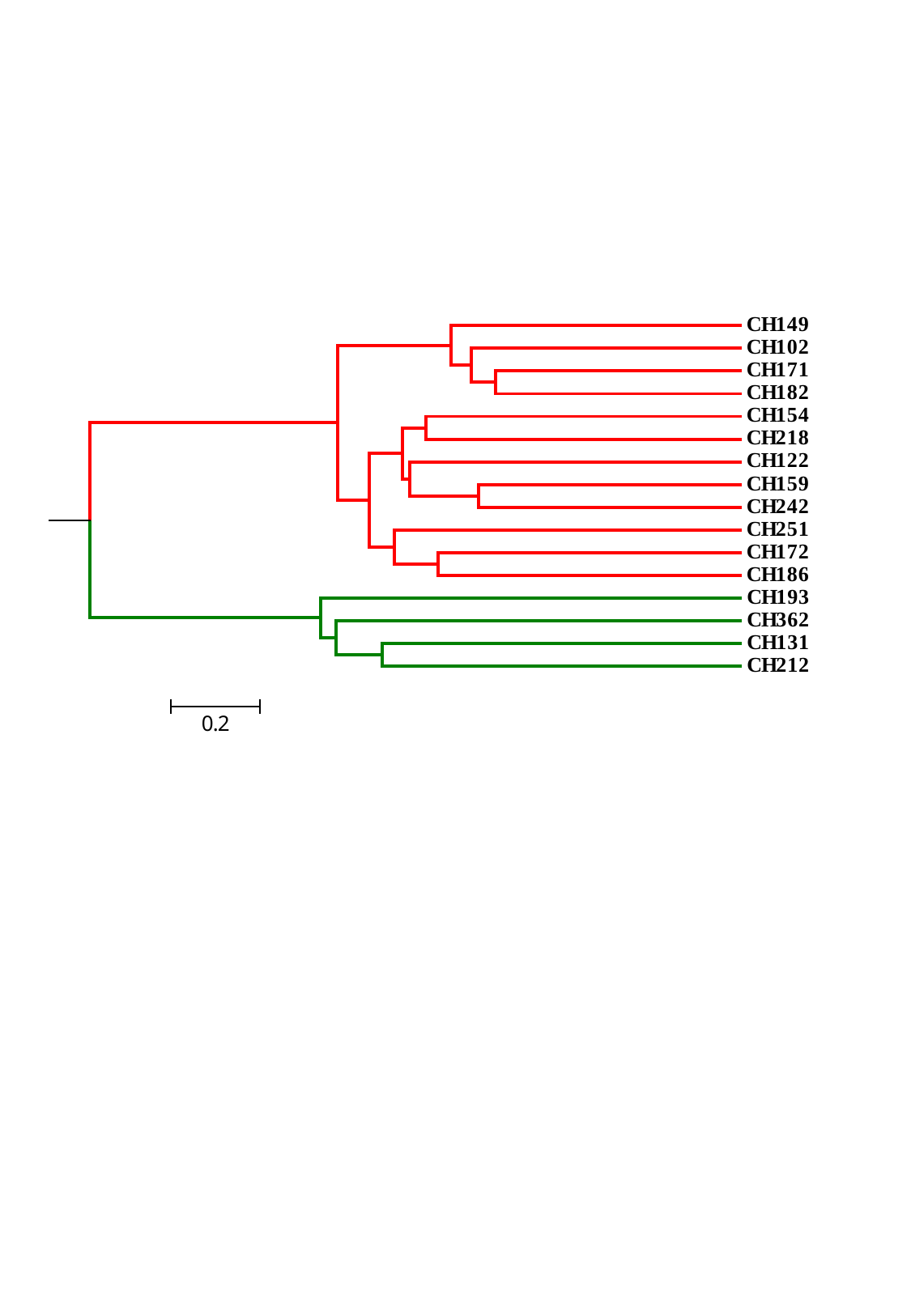

Supplement: Additional file 2: Figure S2 — UPGMA dendrogram of 16 strains based on the SharedAllele distance. [file 12870_2014_311_MOESM2_ESM.ppt]

## Slide 1
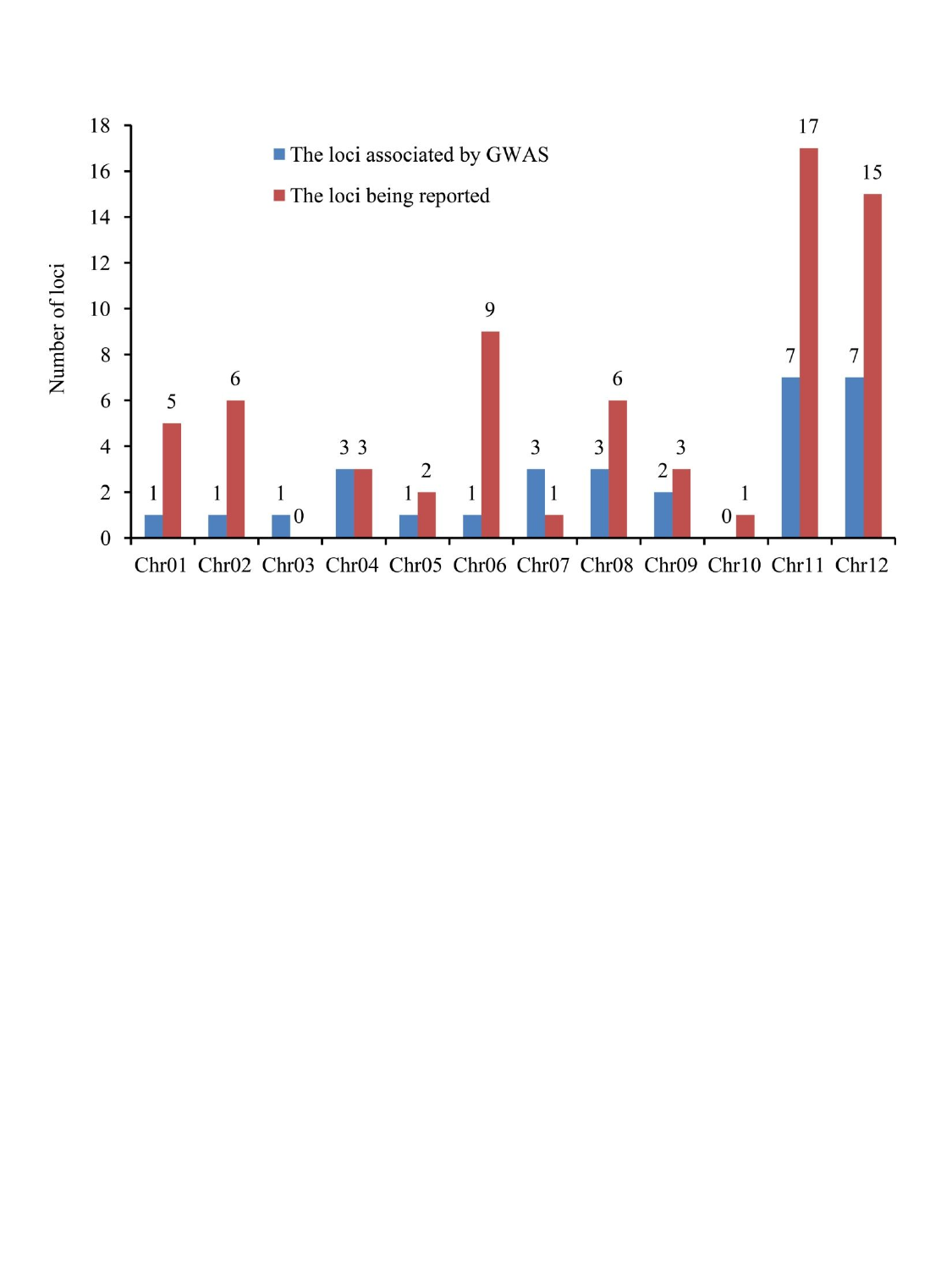

Supplement: Additional file 3: Figure S3 — The number of the associated loci by GWAS in each chromosome. [file 12870_2014_311_MOESM3_ESM.ppt]

## Slide 1
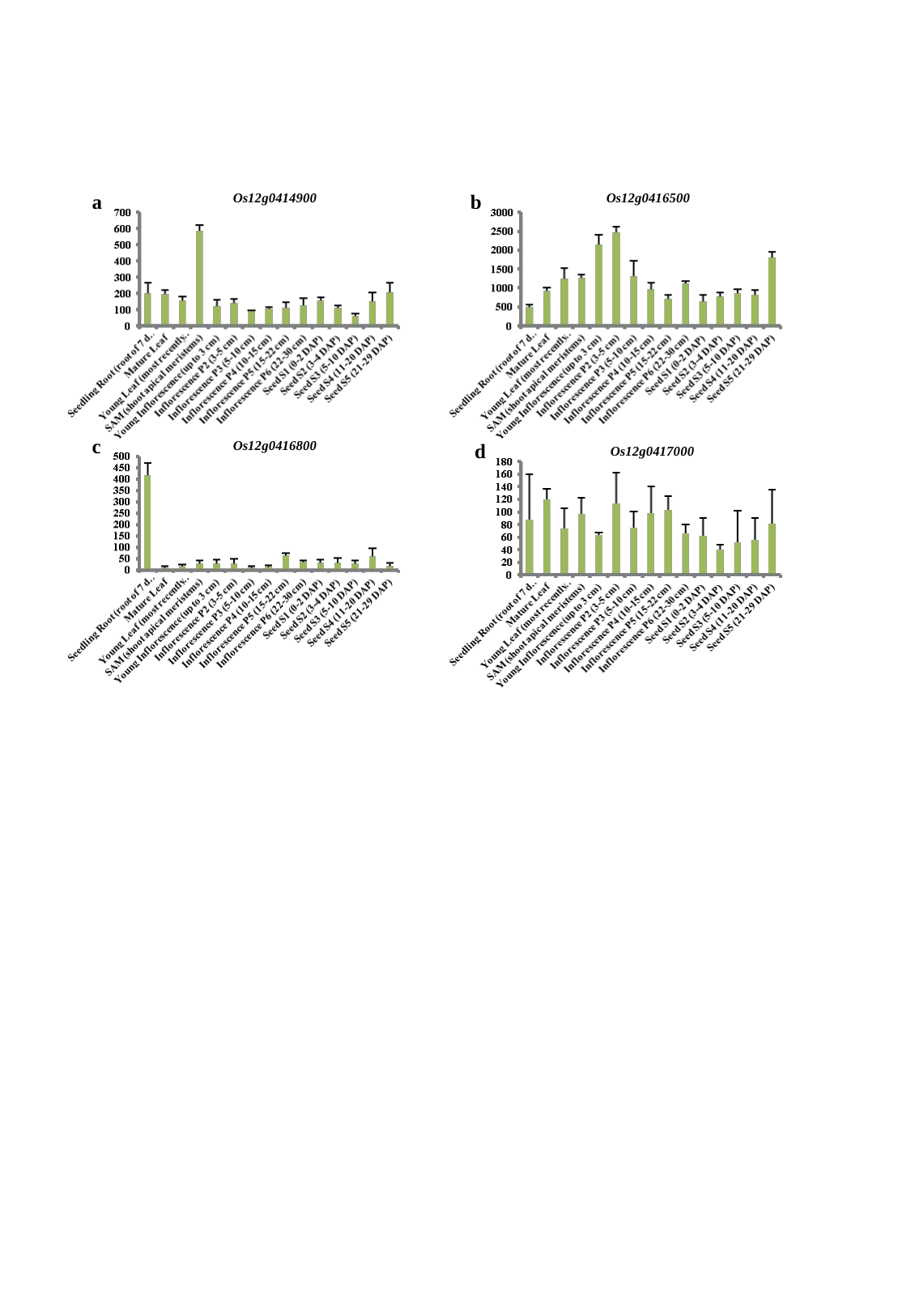

a
Os12g0414900
b
Os12g0416500
c
Os12g0416800
d
Os12g0417000
Figure S6

Supplement: Additional file 8: Figure S6 — Expression pattern analysis of the candidate genes for the associated locus, Chr12_13032951. [file 12870_2014_311_MOESM8_ESM.ppt]
