# Supplementary material for: Genome-wide association study of blast resistance in indica rice
Source: BMC Plant Biol. 2014 Nov 18;14:311. doi: 10.1186/s12870-014-0311-6 (PMC4239320; doi:10.1186/s12870-014-0311-6)
Supplement: Additional file 4: Figure S4 — Functional category annotation for the associated loci by GWAS. [file 12870_2014_311_MOESM4_ESM.ppt]

## Slide 1
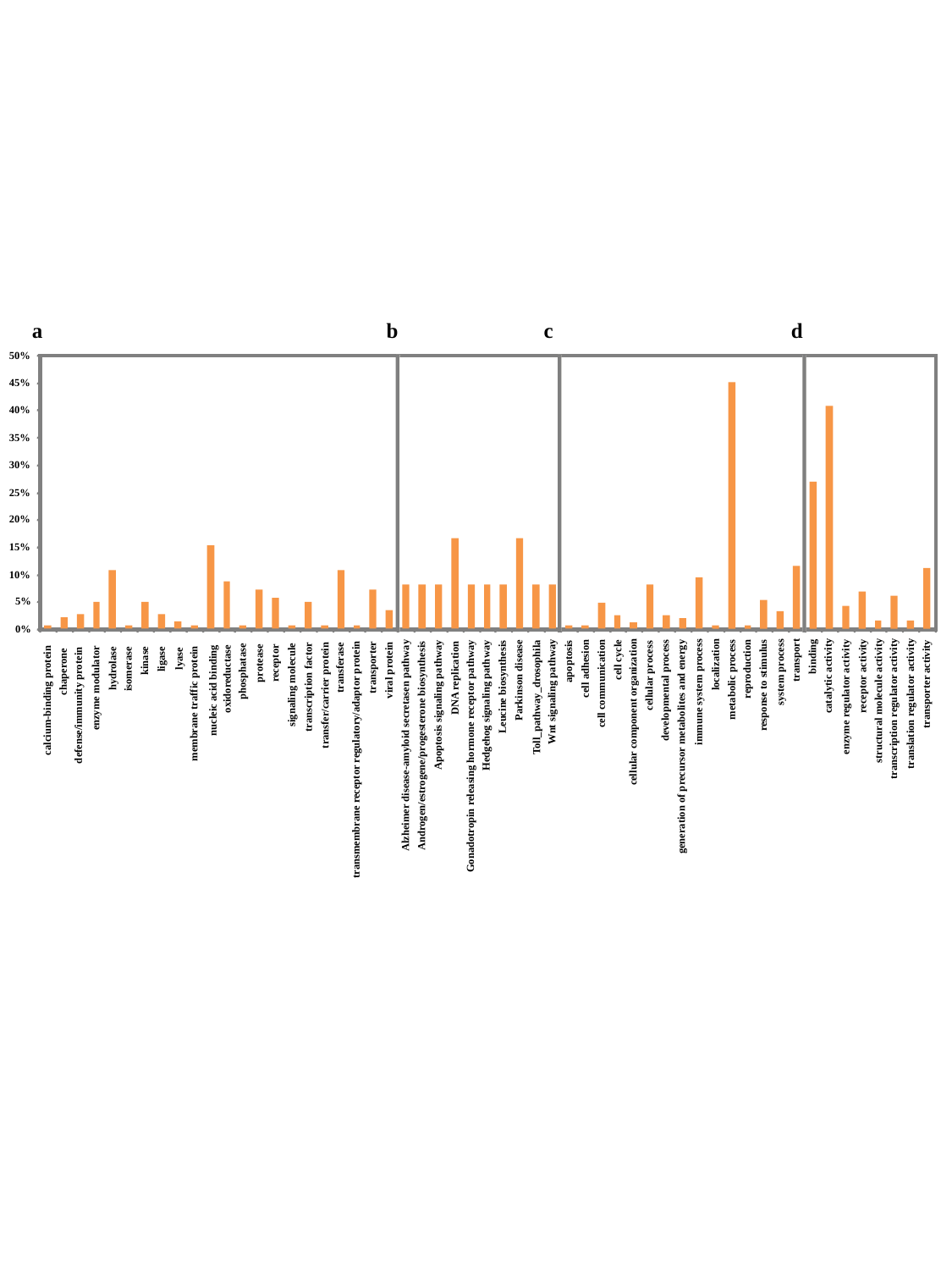

a
b
c
d
50%
45%
40%
35%
30%
25%
20%
15%
10%
5%
0%
binding
lyase
ligase
kinase
cell cycle
transport
apoptosis
receptor
protease
localization
transporter
transferase
hydrolase
isomerase
cell adhesion
viral protein
reproduction
phosphatase
chaperone
system process
cellular process
catalytic activity
receptor activity
DNA replication
oxidoreductase
metabolic process
Parkinson disease
signaling molecule
cell communication
transporter activity
response to stimulus
transcription factor
Leucine biosynthesis
enzyme modulator
nucleic acid binding
developmental process
Wnt signaling pathway
immune system process
transfer/carrier protein
Toll_pathway_drosophila
enzyme regulator activity
calcium-binding protein
structural molecule activity
membrane traffic protein
translation regulator activity
Apoptosis signaling pathway
defense/immunity protein
Hedgehog signaling pathway
transcription regulator activity
cellular component organization
Androgen/estrogene/progesterone biosynthesis
Alzheimer disease-amyloid secretasen pathway
generation of precursor metabolites and energy
Gonadotropin releasing hormone receptor pathway
transmembrane receptor regulatory/adaptor protein
