# Supplementary material for: Genome-wide association study of blast resistance in indica rice
Source: BMC Plant Biol. 2014 Nov 18;14:311. doi: 10.1186/s12870-014-0311-6 (PMC4239320; doi:10.1186/s12870-014-0311-6)
Supplement: Additional file 5: Table S1 — Primers used for quantitative real-time PCR. [file 12870_2014_311_MOESM5_ESM.doc]

**Table S1** Primers used for quantitative real-time PCR

| **Gene** | **Forword Primer (5'-3')** | **Reverse Primer (5'-3')** |
| --- | --- | --- |
| *β*-lectin | CTGCGGGTATCCATGAGA | TACCACCACTGAGAACGATG |
| Os11g0225100 | TGCTGGTCCGCAACTACAC | ACTGGTTCTACAAATCCC |
| Os11g0704100 | GGAGTTTGAGGAGTGGGAGTGGGAT | AACTTGCCCCACTCAGGACCAGATT |
| Os12g0424700 | AGTCGTGGCCGGGCTTC | CGGGCGGCGTGTCAGC |
| Os12g0427000 | GAAGAAGAAGGCGCAAAGCAA | GGACATCCCAAGGTCGCAGA |
| Os12g0414900 | ACAGTATGCTATCAGCCAACCG | CCACAAGCCAACTCTACCAAGC |
| Os12g0415400 | ATGAAGGTGGCACGCAAGTC | TAGCAGCTCTGTGCTCTTCTGG |
| Os12g0415800 | ATGAAGGTGGCACGCAAGTC | GCTCTGGAAGCGAACATAGGAC |
| Os12g0416300 | TCTCATAGTGATGCAAAT | TCTGATTACTCGTGGTCT |
| Os12g0416500 | ACGAATCCCTCCGTCAGCG | CTCGTTGATGTAGAGCTCCTGCA |
| Os12g0416800 | CCCAGAAGCCACCCAAGAAA | TCGTCAACTGCCCTAACAACC |
| Os12g0416900 | CAGCAGTAGGCAACAAAATACAATG | ACCCTCGTAGAAAGCCCTCACA |
| Os12g0417000 | CCATAAGAGGCAAGGCAAAT | TAGGAGGACGCCCAGTTT |
| Os12g0417100 | GTGAGGCAACGGCACAGA | TACCGCCGGTCTTCTCCA |
| Os12g0417600 | GTGACGACGATAATGAGG | AAACTTTGGCTTGCTGTG |
