# Supplementary material for: Genome-wide association study of blast resistance in indica rice
Source: BMC Plant Biol. 2014 Nov 18;14:311. doi: 10.1186/s12870-014-0311-6 (PMC4239320; doi:10.1186/s12870-014-0311-6)
Supplement: Additional file 6: Table S2 — Materials used for quantitative real-time PCR. [file 12870_2014_311_MOESM6_ESM.doc]

**Table S2** Materials used for *q*RT-PCR

| **Strain** | **Resistance/susceptibility** | **Name** | **Resistant rate (%)** |
| --- | --- | --- | --- |
| CH149 | Resistance | Yizhuai(YZA) | 87.5 |
|  | Susceptibility | Laotaigu(LTG) | 25 |
| CH182 | Resistance | Honggeda(HGD) | 62.5 |
|  | Susceptibility | Dachangmangzaodao(DCMHD) | 12.5 |
| CH186 | Resistance | Jinuo(JN) | 87.5 |
|  | Susceptibility | Xianshuichi(XSC) | 6.25 |
| CH362 | Resistance | Xiaohongguzi(XHGZ) | 62.5 |
|  | Susceptibility | Magu(MG) | 12.5 |
