# Supplementary material for: Genome-wide association study of blast resistance in indica rice
Source: BMC Plant Biol. 2014 Nov 18;14:311. doi: 10.1186/s12870-014-0311-6 (PMC4239320; doi:10.1186/s12870-014-0311-6)
Supplement: Additional file 7: Figure S5 — Quantitative real-time PCR analysis of the candidate genes for the associated locus, Chr12_13032951. [file 12870_2014_311_MOESM7_ESM.ppt]

## Slide 1
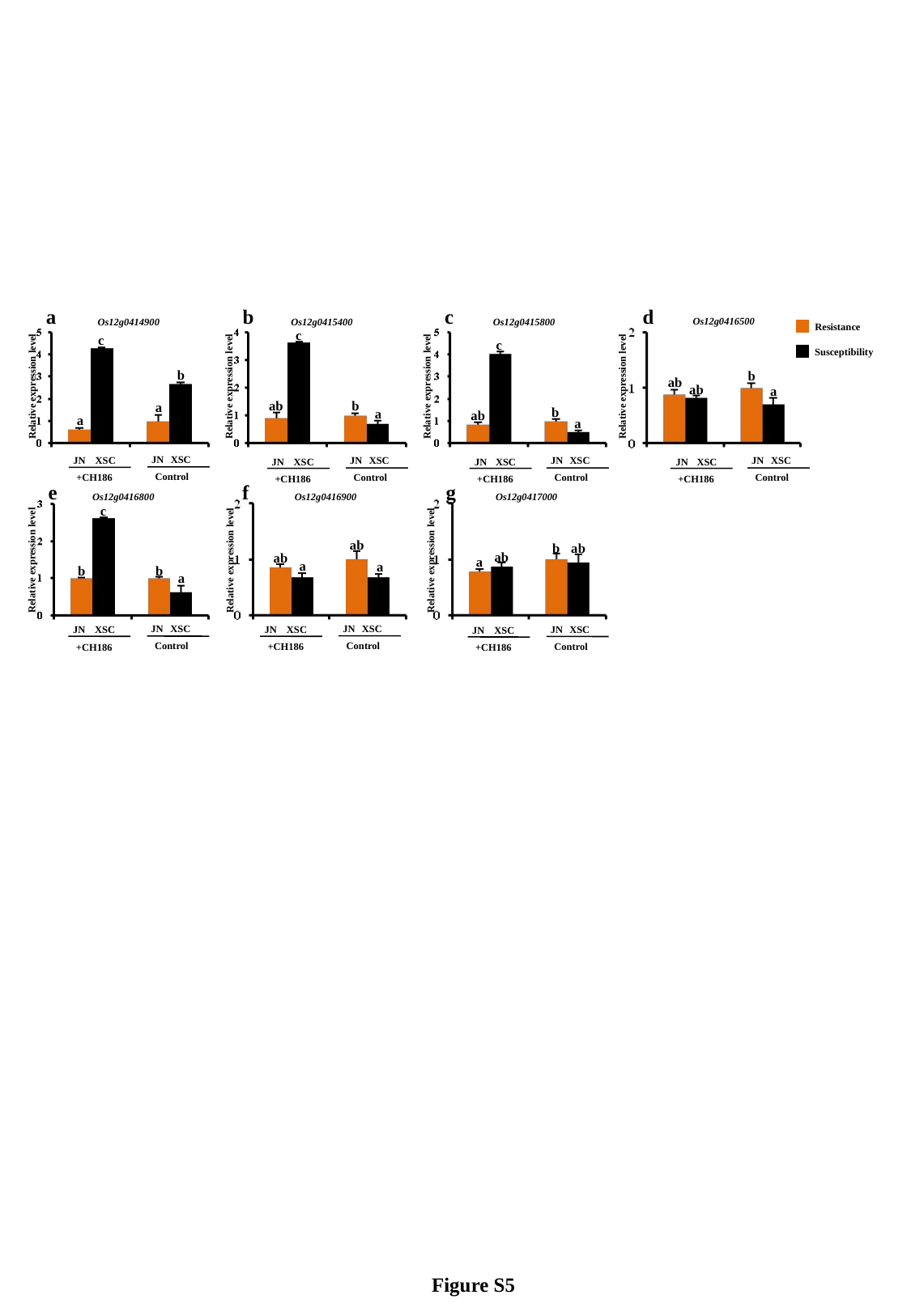

a
b
c
d
Resistance
Os12g0416500
Os12g0414900
Os12g0415400
Os12g0415800
c
c
c
Susceptibility
b
b
ab
ab
a
Relative expression level
Relative expression level
Relative expression level
Relative expression level
ab
b
a
b
a
ab
a
a
JN
XSC
JN
XSC
JN
XSC
JN
XSC
JN
XSC
JN
XSC
XSC
XSC
JN
JN
Control
+CH186
Control
Control
Control
+CH186
+CH186
+CH186
e
f
g
Os12g0416800
Os12g0416900
Os12g0417000
c
ab
b
ab
ab
ab
a
a
a
Relative expression level
Relative expression level
Relative expression level
b
b
a
JN
XSC
JN
XSC
JN
XSC
JN
XSC
JN
XSC
JN
XSC
Control
Control
Control
+CH186
+CH186
+CH186
Figure S5
