# Supplementary material for: Genome-wide association study of blast resistance in indica rice
Source: BMC Plant Biol. 2014 Nov 18;14:311. doi: 10.1186/s12870-014-0311-6 (PMC4239320; doi:10.1186/s12870-014-0311-6)
Supplement: Additional file 9: Table S3 — Summary of 20 candidate genes for Chr03_1170958. [file 12870_2014_311_MOESM9_ESM.doc]

**Table S3 Summary of 20 candidate genes for Chr03_1170958**

| Candidate gene | Annotation description |
| --- | --- |
| *Os03g0119966* | Similar to OsNAC8 protein |
| *Os03g0120000* | Hypothetical protein |
| *Os03g0120100* | Protein of unknown function DUF284 transmembrane eukaryotic family protein. |
| *Os03g0120200* | Remorin C-terminal region domain containing protein |
| *Os03g0120300* | Protein of unknown function DUF1084 family protein |
| *Os03g0120400* | Heavy metal transport/detoxification protein domain containing protein |
| *Os03g0120501* | Non-protein coding transcript uncharacterized transcript |
| *Os03g0120600* | Conserved hypothetical protein |
| *Os03g0120800* | Hypothetical protein |
| *Os03g0120900* | Similar to AP2 domain containing protein RAP2.8 (Fragment) |
| *Os03g0121200* | Similar to Peroxidase 1 |
| *Os03g0121300* | Similar to Peroxidase 1 |
| *Os03g0121700* | Similar to Nascent polypeptide-associated complex alpha subunit-likeprotein 3 (NAC-alpha-like protein 3) (Alpha-NAC-like protein 3) |
| *Os03g0121800* | Double-stranded RNA binding domain containing protein |
| *Os03g0122000* | Protein kinase-like domain containing protein |
| *Os03g0122200* | Similar to 50S ribosomal protein L11 |
| *Os03g0122300* | Similar to Flavanone 3-hydroxylase-like protein |
| *Os03g0122400* | Conserved hypothetical protein |
| *Os03g0122500* | Non-protein coding transcript uncharacterized transcript |
| *Os03g0122600* | Transcription factor MADS-box domain containing protein |
